# Correlation of Immunoglobulin G Expression and Histological Subtype and Stage in Breast Cancer

**DOI:** 10.1371/journal.pone.0058706

**Published:** 2013-03-12

**Authors:** Baokai Yang, Changchun Ma, Zhengshan Chen, Weining Yi, Michael A. McNutt, Yun Wang, Christine Korteweg, Jiang Gu

**Affiliations:** 1 Department of Pathology, School of Basic Medical Sciences, Peking (Beijing) University Health Science Center, Beijing, China; 2 Guangdong Provincial Key Laboratory of Infectious Diseases and Molecular Immunopathology, Department of Pathology and Pathophysiology, Shantou University Medical College, Shantou, China; 3 Department of Radiation Oncology, Cancer Hospital, Shantou University Medical College, Shantou, China; 4 Department of Epidemiology and Biostatistics, School of Public Health, Peking (Beijing) University Health Science Center, Beijing, China; 5 Laboratory of Molecular Pathophysiology, National Institute of Mental Health, National Institutes of Health, Bethesda, Maryland, United States of America; Health Canada, Canada

## Abstract

**Introduction:**

Recently, growing evidence indicates that immunoglobulins (Igs) are not only produced by mature B lymphocytes or plasma cells, but also by various normal cells types at immune privileged sites and neoplasm, including breast cancer. However, the association of breast cancer derived IgG with genesis and development of the disease has not yet been established.

**Methods:**

In this study we examined the expression of IgG in 186 breast cancers, 20 benign breast lesions and 30 normal breast tissues. Both immunohistochemistry with antibodies to Igκ (immunoglobulin G κ light chain) and Igγ (immunoglobulin G heavy chain) and *in situ* hybridization with an antisense probe to IgG1 heavy chain constant region gene were performed. Various clinicopathological features were also analyzed.

**Results:**

We found that IgG is specifically expressed in human breast cancer cells. Both infiltrating ductal carcinoma and infiltrating lobular carcinoma had significantly greater numbers of Igκ and Igγ positive cancer cells as compared with medullary carcinoma, carcinoma in situ, and benign lesions (all p<0.05). In addition, IgG expression was correlated with breast cancer histological subtypes (p<0.01) and AJCC stages (p<0.05), with more abundance of IgG expression in more malignant histological subtypes or in more advanced stage of the disease.

**Conclusions:**

IgG expression in breast cancer cells is correlated with malignancy and AJCC stages of the cancers. This suggests that breast cancer derived IgG may be associated with genesis, development and prognosis of the cancer.

## Introduction

Breast cancer is the most common cancer among women, accounting for 23% of the total cancer cases and 14% of the cancer deaths worldwide. The incidence and mortality of breast cancer is still rising, even though significant progress has been made over the past decades in early diagnosis and treatment [1], [2], [3].

Conventionally, immunoglobulins (Igs) are thought to be produced only by mature B lymphocytes and plasma cells following a complex process of differentiation from precursor B cells. Via gene rearrangement of variable (V), diversity (D) and joining (J) segments B lymphocytes produce Igs in order to recognize and neutralize various pathogens/antigens, and thus contributing to the host humoral immunity. However, recent evidence has demonstrated that human epithelial cancer cells, including cancers of colon, esophagus, breast, nasopharynx, lung, liver, prostate and uterine cervix can also produce immunoglobulins [4], [5], [6], [7], [8], [9].

As to Ig expression in breast cancers, Qiu et al. was the first to demonstrate IgG synthesis in purified breast cancer cells and a breast cancer cell line (MCF-7) with immunohistochemistry (IHC), *in situ* hybridization (ISH) and Western blot [6]. In addition, they found that blocking tumor-derived IgG by either antisense DNA or anti-IgG antibody could increase apoptosis and growth inhibition of cancer cells *in vitro* [6]. Chen et al. further confirmed the IgG expression in breast cancer by IHC, ISH, and laser microdissection followed by reverse transcription-polymerase chain reaction (RT-PCR) [4], [5]. In addition, Babbage et al. amplified VH gene transcripts by nested RT-PCR as either single or dual V(D)J rearrangements in four of six breast cancer cell lines [10]. Furthermore, activation-induced cytidine deaminase, an enzyme which is required for both class switch recombination (CSR) and somatic hypermutation (SHM) in B lymphocytes, was found to be expressed in six breast cancer cell lines [10]. These studies together established the capacity of breast carcinomas to endogenously produce IgG. However, the association of breast cancer derived IgG with genesis and development of the disease has not yet been established. The biologic impact of cancer cell-derived IgG on breast cancer is not yet clear.

In this study, we investigated the expression of IgG in breast cancer tissues, benign lesions and normal breast tissues of breast with IHC and ISH. In addition, the relationships between IgG expression levels and various clinicopathological features of breast cancer were analyzed.

## Methods

### Sample collection

Formalin-fixed, paraffin-embedded breast tissues were obtained from 236 patients in the archives of The 252nd Hospital of the Peoples' Liberation Army (Bao Ding, China), and were collected from 1997 to 2005. These tissue specimens included 186 breast cancers, 20 benign lesions (10 fibroadenomas and 10 breast hyperplasias) and 30 normal breast tissues (11 from normal female breast tissue surrounding benign lesions, 19 from gynecomastia). Clinical data from these 186 breast cancer patients were obtained, and the characteristics of these patients are detailed in Table 1. Tumor size was determined by measurement of the excised lesion and only the largest tumor diameter was used for analysis. Histological typing of breast cancers was based on the 2003 WHO classification of tumors [11]. The staging of breast cancer was based on the sixth edition of the American Joint Committee on Cancer (AJCC) Cancer Staging Manual [12]. Histological grading of breast cancer was determined according to the Scarff-Bloom-Richardson (SBR) method [13]. Histological typing and grading was performed by pathologists of The 252nd Hospital of the PLA and re-evaluated by two independent pathologists from the Peking University Health Science Center. Inter-examiner discrepancies were resolved by joint examination and mutual consensus of the two independent pathologists. The study was approved by the Ethical Committee of Medical Health Sciences Center of Peking University and written consent was obtained from the patients.

**Table 1:** Characteristics of patients and their breast cancers.

Characteristics	No. of patients	%
Age		
<40	16	8.6
40–50	64	39.8
51–70	96	46.2
>70	10	5.4
Histology		
ILC	28	15.1
IDC	127	68.3
Medullary	16	8.6
DCIS/LCIS	15	8.1
AJCC Stage		
0	14	7.5
1	14	7.5
2	99	53.3
3	59	31.7
Axillary node		
0	82	44.6
1–3	44	23.1
>3	60	32.3
Tumor size (cm)		
0–0.9	2	1.1
1–1.9	21	10.8
2–2.9	45	23
>3	118	65.1
SBR Score		
1	21	11.4
2	60	33.3
3	90	47.3
Estrogen receptor		
Positive	94	50.5
Negative	92	49.5
Progesterone receptor		
Positive	82	44.1
Negative	104	55.9
CerbB-2		
Positive	58	31.2
Negative	128	68.8
PCNA		
High(>47)	111	59.7
Low(≤47)	75	40.3
p53		
Positive	69	37.1
Negative	117	62.9
Nm23		
High	81	43.5
Low	105	56.5

Abbreviations: ILC, Infiltrating lobular carcinoma; IDC, infiltrating ductal carcinoma;

MC, medullary carcinoma; CIS, carcinoma in situ; DCIS/LCIS, ductal carcinoma *in situ*/

Lobular carcinoma *in situ*; AJCC, American Joint Committee on Cancer;

SBR, Scarff-Bloom-Richardson.

### Immunohistochemistry

IHC was performed as previously described [4]. In short, 4 µm-thick sections were deparaffinized and rehydrated in graded concentrations of ethanol to distilled water. Sections were placed into 10 mM citrate buffer (pH 6.0), heated in a microwave oven at 95°C for 15 min, and then cooled to room temperature. After rinsing with PBS, the sections were inserted in 3% hydrogen peroxide (H_2_O_2_) for 30 min and rinsed again in 0.01 M PBS at room temperature. Before applying primary antibodies, sections were blocked with 10% normal sheep serum for 30 min. The primary antibody, rabbit anti-human IgG heavy chain (Igγ) (1∶1000; Dako) or mouse anti-human IgG kappa light chain (Igκ) (1∶500; Zymed Laboratories) was added and incubated overnight at 4°C. Polymer detection system for immune-histological staining (Zymed Laboratories) was used, which gives a red color. Sections were then counterstained with hematoxylin. Primary antibody was replaced by PBS as a negative control. A pre-absorption test was also performed as a negative control with pre-incubation of anti-human Igγ antibody (Dako) and standard human IgG (3 and 20-fold concentration of working primary antibody respectively, Sigma http://en.wikipedia.org/wiki/Wikipedia:IPA_for_English) at 4°C, overnight. In addition, replacing anti-human Igγ antibody with normal rabbit IgG (Santa Cruz) or normal mouse IgG (Santa Cruz) was used as negative control. Normal human tonsil tissue was used as positive control. In case of B lymphocytes infiltrating in cancer tissues, such cells also served as a positive internal control. Details of primary antibodies to Igγ Igκ, estrogen receptor (ER), progesterone receptor (PR), non-metastatic 23 protein (nm23), P53, proliferating cell nuclear antigen (PCNA), human epidermal growth factor receptor 2 (HER2/neu), CD20, CD68, cytokeratin (CK), and Smooth Muscle Actin (SMA) were listed in Table 2. The Destain & restain technique was performed as described previously [14].

**Table 2:** Primary antibodies and detection system used.

Primary antibody and detection system	Catalog #	Suplier
rabbit anti-human IgG heavy chain (Igγ)	A0423	Dako, Carpinteria, CA
mouse anti-human IgG kappa light chain (Igκ)	180031	Zymed Laboratories, South San Francisco, USA
mouse anti-human estrogen receptor (ER)	491002	Zymed Laboratories, South San Francisco, USA
mouse anti-human progesterone receptor (PR)	419500	Zymed Laboratories, South San Francisco, USA
mouse anti-human non-metastatic 23 protein (nm23)	PN117796	Zymed Laboratories, South San Francisco, USA
mouse anti-human P53	AHO0152	Zymed Laboratories, South San Francisco, USA
mouse anti-human proliferating cell nuclear antigen (PCNA)	180110	Zymed Laboratories, South San Francisco, USA
mouse anti-human human epidermal growth factor receptor 2 (HER2/neu)	44798G	Zymed Laboratories, South San Francisco, USA
mouse anti-human CD20	ZM-0039	ZSGB-BIO, Beijing, China
mouse anti-human CD68	180491	Zymed Laboratories, South San Francisco, USA
mouse anti-human cytokeratin (CK)	ZM-0069	ZSGB-BIO, Beijing, China
mouse anti-human Smooth Muscle Actin (SMA)	180106	Zymed Laboratories, South San Francisco, USA
standard human IgG	56834	Sigma-Aldrich Corporation, St. Louis, USAhttp://en.wikipedia.org/wiki/Wikipedia:IPA_for_English
normal rabbit IgG	sc-2027	Santa Cruz Biotechnology, Inc., California, USA
normal mouse IgG	sc-2025	Santa Cruz Biotechnology, Inc., California, USA
Polymer detection system for immune-histological staining	879983	Zymed Laboratories, South San Francisco, USA
anti-digoxigenin antibody (Fab fragment) conjugated with alkaline phosphatase	11093274910	Roche Diagnostics, Indianapolis, IN, USA
5-Bromo-4-chloro-3-indolyl phosphate and nitro-blue-tetrazolium	B3804	Sigma, St Louis, MO, USA

### *In situ* hybridization

ISH was performed on tissue sections consecutive to sections showing Igγ positive cells as identified by IHC. In brief description, 4 µm-thick sections were deparaffinized and dehydrated, incubated in 0.1 M HCl for 10 min, heated to 95°C in 10 mM citrate buffer (pH 6.0) using a microwave oven for 20 min, cooled to room temperature, washed in PBS, and then fixed in 4% paraformaldehyde for 10 min. After dehydrating again with 90% ethanol for 15 sec, sections were incubated with a hybridization cocktail [15] containing digoxigenin-labeled cRNA probe of human antisense or sense immunoglobulin G1 heavy chain (IGHG1) at 42°C overnight, washed in 2xSSC plus 50% formamide once for 15 min and in 2xSSC twice for 15 min at 37°C. Sections were incubated with horse serum at room temperature for 60 min, and then incubated with anti-digoxigenin antibody (Fab fragment) conjugated with alkaline phosphatase (Roche Diagnostics, Indianapolis, IN, USA). 5-Bromo-4-chloro-3-indolyl phosphate and nitro-blue-tetrazolium (Sigma, St Louis, MO, USA) were finally used to visualize the signals [5]. Normal human tonsil served as the positive control. Sections incubated with the corresponding sense probe were used as negative control.

### Score of Igκ and Igγ IHC staining in breast cancer

Microscopic evaluation was performed in a blinded fashion by two pathologists on a minimum of 10 randomly selected fields using a 40× objective lens and a total of 1000 cells per slides were counted. Discrepancies were resolved as aforementioned. The percentages of positive cancer cells to the total ones were calculated. For Igκ and Igγ, each sample was scored for intensity of signal (0 = none, 1 = weak, 2 = moderate, 3 = strong) and percentage of positive cells (0 = none, 1 = <10%, 2 = 10–25%, 3 = 25–50%, 4 = >50%). The terms focal and diffuse are defined for purposes of this study, as focal (1) less than or equal to 25%, and diffuse (2) greater than 25%. The scoring of staining positivity and semi-quantitative analysis of the morphological data were adapted from the commonly employed criteria reported in the literature [16] and adjusted to this application. For evaluation of ER, PR, p53 and HER2/neu staining, only the percentage of positive cells was recorded. For purpose of analysis, cut-off points were chosen to divide the cases into ‘positive’ and ‘negative’ groups. A 10% cut-off point was chosen for ER, PR and, and 25% was chosen as the cut-off point for HER2/neu [17]. High nm23 was defined as expression of nm23 by all cells. If there were any unstained tumor cells, the tumor was scored as low nm23. Some variability in staining intensity of nm23 was noted. However, the system of scoring used in this study was based on the proportion of stained cells only, as has been previously described for scoring of nm23 [18], [19]. For PCNA, the percentage of positive stained cells recorded as the PCNA labeling index (PCNA-LI) was calculated as follows: LI = 100 p/t, where LI, represents labeling index; p, the number of positive cells counted; and t, the total number of (positive and negative) cells counted [20]. The mean value of the PCNA-LI was then used as a cut-off point to divide all cases into 2 groups with high and low indices. In this study, the PCNA labeling indices ranged from 0 to 95%, with a mean value of 47.46%. The cut-off point 47%, which is close to the cut-off used in a previous report of 45% [21] was chosen. 111 tumors had a high PCNA-LI of >47% and 75 tumors had a low PCNA-LI of ≤47%.

### Statistical analyses

IgG expression among human breast cancers, benign breast lesions and normal breast tissues were compared using the Kruskal-Wallis test [21]. When the test was significant (p<0.05), pairwise multiple comparison procedures (Dunn's Method) [22] were then used, and the p-values were adjusted by the Bonferroni method [23]. In order to evaluate the correlation of IgG with AJCC stage, SBR score, tumor size, histological subtype, age, ER, PR, HER2/neu, p53, PCNA and nm23, Kendall's Tau-b assay was used [23].

## Results

### Both IgG protein and IGHG1 mRNA are expressed in human breast cancer cells

**Figure 1:**
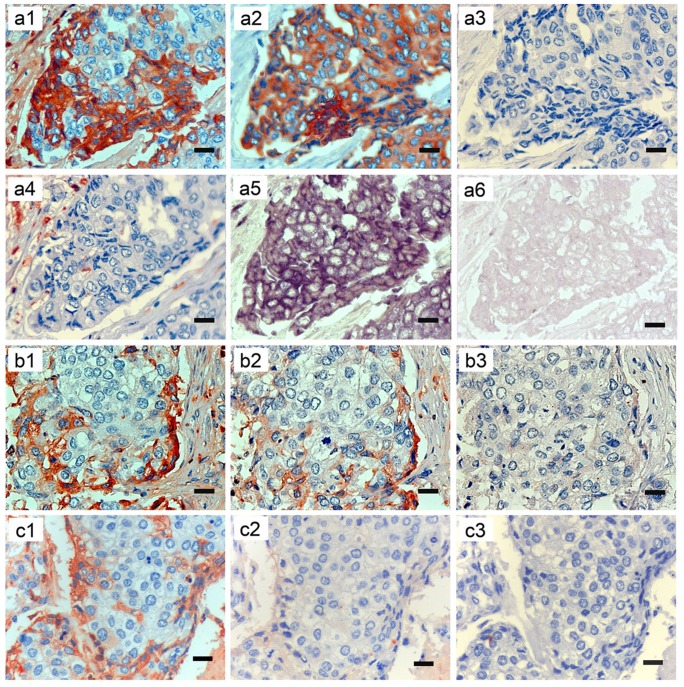
Human breast cancer expresses Igγ protein and IGHG1 mRNA as detected by IHC and ISH. a1–6, b1–3 and c1–3 are serial sections of breast cancer tissue (IDC) respectively. a1–4 showing IHC with antibodies to to Igγ (IgG heavy chain, a1), CK (an epithelial cell marker, a2), CD20 (a B cell marker, a3) and CD68 (a macrophage marker, a4). a5–6 showing ISH with an IGHG1 mRNA antisense probe (a5), and with a sense probe (a6). b1–3 showing IHC with antibody to Igγ alone, antibody to Igγ preincubated with standard human IgG of 3-fold and 20-fold concentration of the working primary antibody respectively. c1–3 showing IHC staining after incubation with antibody to Igγ, normal rabbit IgG and normal mouse IgG respectively, without positive signals in the Igγ expressing cells in the latter two sections. Original magnifications: ×400. Scale bars: 20 µm.

IHC with antibodies to Igγ, CK, CD20 and CD68 was carried out on serial sections of breast cancer tissue (invasive ductal carcinoma) (Fig. 1, a1–4). Igγ showed positive staining in the cytoplasm of breast cancer cells, mainly located in the periphery of the tumor nest (a1). Positive staining for CK demonstrated that the Igγ positive cells were of epithelial cell origin (a2), whereas negative staining for CD20 excluded the possibility that these cells were infiltrating B lymphocytes (a3). IHC with antibody to CD68 showed that macrophages were mainly located in the interstitial tissue (a4). Furthermore, in order to confirm that positive Igγ expression was actually the result of Igγ production by these breast cancer cells, ISH with an antisense probe to IGHG1 was performed on serial sections. Positive signals were also distributed in the cytoplasm of breast cancer cells (a5). Application of an IGHG1 sense probe as negative control did not show any positive signal (a6). The number of IGHG1 mRNA expressing breast cancer cells is much larger than that of Igγ expressing ones. A preabsorption test showed normal Igγ staining, weaker staining (preincubated with standard human IgG of 3-fold concentration of primary antibody) and no Igγ staining (preincubated with standard human IgG of 20-fold concentration of primary antibody) on serial sections of breast cancer (Fig. 1, b1–3). Incubated with antibody to Igγ, normal rabbit IgG and normal mouse IgG respectively on serial sections of breast cancer, the last two showed no IHC staining positive signals in the Igγ expressing cells (Fig. 1, c1–3).

### Igκ and Igγ are expressed at different levels among cancers, benign lesions and normal tissues of human breast

Igκ and Igγ staining of positive controls (human normal tonsil) or infiltrating lymphocytes in breast cancer specimens appeared intensely dark red indicating abundant expression of IgG. Igκ and Igγ signal intensity were scored on a scale of 0 to 3 as described above. No case was found to express these proteins as strongly as the positive controls. Of 127 infiltrating ductal carcinomas (IDC), a total of 118 (92.9%) scored positively for expression of Igκ (Table 3). This included 70 of 127 (55.1%) which showed a score of 2. Only 5 (3.9%) cases had a score of 3. IDC showed diffuse Igγ staining (>25% of cells) in 68 of 127 (53.6%) cases (Table 3) and both intensity and extent of expression of Igγ in IDC was similar to that of Igκ (Table 3).

**Table 3:** Igk and Igγ expression in cancers, benign lesions and normal tissues of human breast.

Factor	Histotype	No. patients	Score, n (%)				
			0	1	2	3	4
Igk percentage	ILC	28	0(0)	0(0)	3(10.7)	10(35.7)	15(53.6)
	IDC	127	9(7.1)	27(21.2)	23(18.1)	28(22.1)	40(31.5)
	MC	16	1(6.2)	10(62.5)	4(25.0)	1(6.3)	0(0)
	CIS	15	4(26.7)	6(40)	4(26.7)	1(6.7)	0(0)
	BL	20	7(35)	6(30)	4(20)	3(15)	0(0)
	NB	30	5(16.7)	19(63.3)	5(16.7)	1(3.3)	0(0)
Igγ percentage	ILC	28	1(3.6)	0(0)	3(10.7)	10(35.7)	14(50.0)
	IDC	127	12(9.4)	28(22.1)	30(23.6)	23(18.1)	34(26.8)
	MC	16	1(6.2)	9(56.3)	5(31.3)	1(6.2)	0(0)
	CIS	15	3(20.0)	10(66.7)	1(6.7)	1(6.7)	0(0)
	BL	20	7(35.0)	7(35.0)	4(20.0)	2(10.0)	0(0)
	NB	30	7(23.3)	17(56.7)	5(16.7)	1(3.3)	0(0)
Igk intensity	ILC	28	0(0)	2(7.1)	25(89.3)	1(3.6)	
	IDC	127	9(7.1)	43(33.9)	70(55.1)	5(3.9)	
	MC	16	1(6.2)	7(43.8)	8(50.0)	0(0)	
	CIS	15	4(26.7)	4(26.7)	7(46.6)	0(0)	
	BL	20	7(35.0)	11(55.0)	2(10.0)	0(0)	
	NB	30	5(16.7)	24(80.0)	1(3.3)	0(0)	
Igγ intensity	ILC	28	1(3.6)	2(7.1)	24(85.7)	1(3.6)	
	IDC	127	12(9.4)	42(33.1)	68(53.5)	5(3.9)	
	MC	16	1(6.2)	6(37.5)	9(56.3)	0(0)	
	CIS	15	3(20.0)	5(33.3)	7(46.7)	0(0)	
	BL	20	7(35.0)	10(50.0)	3(15.0)	0(0)	
	NB	30	7(23.3)	22(73.4)	1(3.3)	0(0)	

**NOTE:** Percentage scores of Igκ and Igγ (based on the percentage of tumor cells stained in the sample): 0 = none, 1 = <10%, 2 = 10 to 25%, 3 = 25 to 50%, 4 = >50%. The staining intensity scores of Igκ and Igγ (according to positive cytoplastic signals): 0 = none, 1 = weak, 2 = moderate, 3 = strong.

Abbreviations: IHC, immunohistochemistry; ILC, infiltrating lobular carcinoma; IDC, infiltrating ductal carcinoma; MC, medullary carcinoma; CIS, carcinoma *in situ*; BL, benign lesions of breast; NB, normal breast tissues.

All 28 infiltrating lobular carcinomas (ILC) (100%) scored positively for Igκ expression with 25 of 28 (89.3%) showing an intensity score of 2, and there was only 1 case (3.6%) with a score of 3 (Table 3). Igκ-expressing cells in ILC were found to be diffuse (>25% of cells) in most cases (25 of 28, 89.3%) (Table 3). The expression of Igγ in ILC also was similar to that of Igκ (Table 3).

Medullary carcinoma (MC) and carcinoma in situ (CIS) showed Igκ expression in 15 of 16 (93.8%) and 11 of 15 (73.3%) cases respectively, but neither had any case with an intensity score of 3 (Table 3). Moreover, MC and CIS showed only focal Igκ expression in 14 of 16 (87.5%) and 10 of 15 (66.7%) respectively (Table 3). 14 of 16 (87.5%) cases of MC and 12 of 15 (80%) cases of CIS had weak to moderate intensity of Igγ expression. In 14 of 16 (87.5%) cases of MC and 11 of 15 (74.4%) cases of CIS there was only a focal pattern of expression (Table 3).

**Figure 2:**
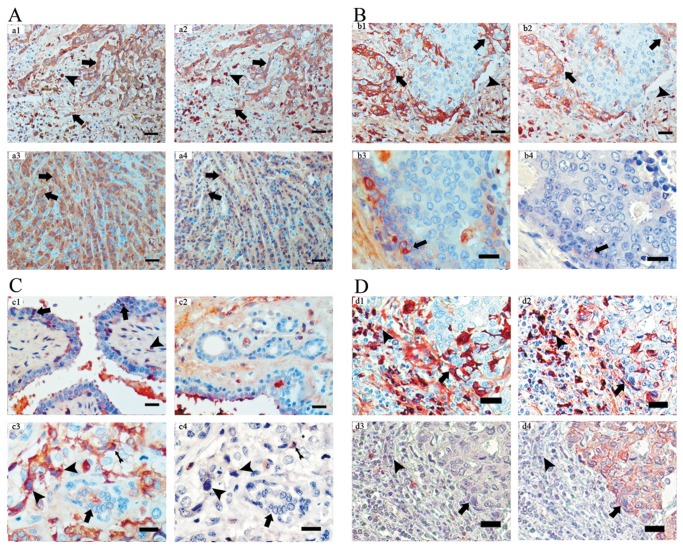
Igκ and Igγ expression in cancers, benign lesions and normal tissues of breast by immunohistochemistry. A: Igγ (a1, a3) and Igκ (a2, a4) are diffusely positive in cell cords and single cell chains in IDC (a1 and a2) and ILC (a3 and a4) (arrowheads) with stronger staining in B lymphocytes/plasma cells (letterheads) in stroma. B: Igγ (b1) and Igκ (b2) are expressed in some cells inside large cell groups of IDC (arrowheads) and infiltrating lymphocytes (letterheads). In DCIS Igγ (b3) and Igκ (b4) expression can be seen in a few cells (arrowheads). C: Igκ positive cells can also be seen in glandular epithelial cells (arrowheads) of fibroadenoma (c1) along lumens and in a limited number of fibroblasts (letterhead) in the interstitium. Igκ expression can seldom been detected in normal breast duct adjacent to benign breast lesion (cystic hyperplasia of breast) (c2). Destain & restain with antibodies to Igκ and CD20 on IDC shows more Igκ expression in infiltrating cancer cells (c3, letterheads) than in adjacent histologically normal breast duct (c3, arrowhead), with infiltrating B lymphocytes as internal positive control (costaining with both Igκ and CD20) (c4, black arrow). D: By conducting immonostaining and destain & restaining with antibodies against Igγ, Igκ, CD20 and CK on two consecutive sections of axillary lymph node metastatic breast cancer tissue, we observed costaining of Igγ and Igκ in both breast cancer cells (d1 and d2) in cancer nest (CK positive) and stronger staining in B lymphoid/plasma cells (d1 and d2, letterheads) in tumor stroma. The lymphoid/plasma cells with or without CD20 staining (d3, letterhead) in tumor stroma showed no positive CK staining with breast cancer cells as internal positive control (d4, arrowheads). Original magnifications: a1–a4, b1 and b2 ×200, scale bars: 40 µm; others ×400, scale bars: 20 µm.

Most benign lesions of breast and normal breast tissues had weak and only focal expression of Igκ and Igγ (Table 3). In benign fibroadenomas, 8 of 10 (80%) cases expressed Igκ and Igγ, which was found not only in glandular epithelia but also in interstitial cells such as fibroblasts. Breast hyperplasia showed expression of Igκ and Igγ in 5 of 10 cases (50%). In normal breast tissues, Igκ and Igγ were positive in 25 of 30 (83.3%) and 23 of 30 (76.4%) cases respectively. However, more than 50% of cases of normal breast tissue had only focal expression of Igκ and Igγ. Fig. 2 (A–D) demonstrates the expression of Igκ and Igγ in cancers, benign lesions and normal breast tissues.

According to the statistical analysis, both IDC and ILC showed significantly (all p<0.05) greater signal intensity in Igκ and Igγ as compared with benign lesions and normal tissues (Table 3). However, Igκ and Igγ had significantly higher staining intensity as compared with CIS only in ILC, and not in IDC (Table 4). ILC also showed greater Igκ signal intensity than MC but this was not true for Igγ signal intensity (Table 4). IDC showed no difference when compared either with MC or CIS in Igκ and Igγ staining intensity, and there were no significant differences in Igκ and Igγ signal intensity among MC, CIS, benign lesions and normal tissues (Table 4).

**Table 4:** Multiple comparisons of immunostaining intensity of Igκ and Igγ in benign and malignant breast diseases.

Dependent	Histotype	Histotype	absolute value	Dependent	Histotype	Histotype	absolute value
Variable	(I)	(J)	of z	Variable	(I)	(J)	of z
Igκ intensity	ILC	IDC	2.754	Igγ intensity	ILC	IDC	2.654
		MC	3.282*			MC	2.779
		CIS	3.475*			CIS	2.967*
		BL	5.365*			BL	4.731*
		NB	5.912*			NB	5.681*
	IDC	ILC	2.754		IDC	ILC	2.654
		MC	1.409			MC	0.871
		CIS	1.936			CIS	1.421
		BL	4.088*			BL	3.408*
		NB	4.747*			NB	4.553*
	MC	ILC	3.283*		MC	ILC	2.779
		IDC	1.409			IDC	0.871
		CIS	0.733			CIS	0.645
		BL	2.368			BL	2.194
		NB	2.573			NB	2.854
	CIS	ILC	3.475*		CIS	ILC	2.967*
		IDC	1.936			IDC	1.421
		MC	0.733			MC	0.646
		BL	1.343			BL	1.276
		NB	1.397			NB	1.718
	BL	ILC	5.365*		BL	ILC	4.731*
		IDC	4.088*			IDC	3.408*
		MC	2.368			MC	2.194
		CIS	1.343			CIS	1.276
		NB	0.06			NB	0.373
	NB	ILC	5.912*		NB	ILC	5.681*
		IDC	4.747*			IDC	4.553*
		MC	2.573			MC	2.854
		CIS	1.397			CIS	1.718
		BL	0.06			BL	0.373

* The absolute value of z is significant at the 0.05 level, the critical value of z was 2.935. Abbreviations: ILC, Infiltrating lobular carcinoma; IDC, infiltrating ductal carcinoma; MC, medullary carcinoma; CIS, carcinoma *in situ*; BL, benign lesions of breast; NB, normal breast tissues.

Percentages of Igκ and Igγ in ILC and IDC were significantly greater (all p<0.05) compared with MC, CIS, benign lesions and normal breast tissues (Table 5). Percentages of Igκ and Igγ in ILC and IDC also showed significant differences (p<0.05) (Table 5). There was no statistical difference in the percentages of Igκ and Igγ among MC, CIS, benign lesions and normal tissues (Table 5).

**Table 5:** Multiple comparisons of percentage of Igκ and Igγ positive cells in benign and malignant lesions.

Dependent	Histotype	Histotype	absolute value	Dependent	Histotype	Histotype	absolute value
Variable	(I)	(J)	of z	Variable	(I)	(J)	of z
Igκ percentage	ILC	IDC	3.138*	Igγ percentage	ILC	IDC	3.550*
		MC	5.572*			MC	5.129*
		CIS	5.235*			CIS	5.310*
		BL	5.537*			BL	5.449*
		NB	6.422*			NB	6.400*
	IDC	ILC	3.138*		IDC	ILC	3.551*
		MC	3.928*			MC	2.954*
		CIS	3.692*			CIS	3.463*
		BL	3.962*			BL	3.497*
		NB	5.005*			NB	4.553*
	MC	ILC	5.572*		MC	ILC	5.129*
		IDC	3.928*			IDC	2.954*
		CIS	0.581			CIS	1.026
		BL	0.451			BL	0.767
		NB	0.755			NB	1.183
	CIS	ILC	5.235*		CIS	ILC	5.310*
		IDC	3.692*			IDC	3.463*
		MC	0.581			MC	1.026
		BL	0.158			BL	0.303
		NB	0.039			NB	0.055
	BL	ILC	5.537*		BL	ILC	5.449*
		IDC	3.962*			IDC	3.497*
		MC	0.451			MC	0.767
		CIS	0.158			CIS	0.303
		NB	0.23			NB	0.299
	NB	ILC	6.422*		NB	ILC	6.400*
		IDC	5.005*			IDC	4.553*
		MC	0.755			MC	1.183
		CIS	0.039			CIS	0.055
		BL	0.23			BL	0.299

* The absolute value of z is significant at the 0.05 level, the critical value of z was 2.935.

Abbreviations: ILC, Infiltrating lobular carcinoma; IDC, infiltrating ductal carcinoma;

MC, medullary carcinoma; CIS, carcinoma *in situ*; BL, benign lesions of breast;

NB, normal breast tissues.

### Growth pattern of cancer cells appears to be related to IgG expression

Cancer cells arrayed in scattered small cords, small nests and individually or in cell ‘chains’ showed more positive staining cells than those arrayed in large groups and syncytial sheets (Fig. 2, A and B). The highest positive percentage score was found in cases of ILC, 53.6% (Igκ) and 50% (Igγ), and in these cases most cells were arranged singly or in small cell strands (Table 3). Cells in MC are by definition arranged in large cell groups, and 87.5% (Igκ and Igγ) of cases of MC showed only focal expression (Table 3). IDC with moderately sized cell groups showed expression values between those of MC and ILC.

### Igκ and Igγ expression are significantly correlated with histological types of breast cancer

As shown in Table 6, the expression of Igκ and expression of Igγ in breast cancer were significantly correlated (all p<0.01). Igγ and Igκ expressions also showed significant association with histological subtypes of breast carcinoma (all p<0.01) and AJCC stage (all p<0.05). However, it was of interest that only the signal intensity scores of Igκ and Igγ and not positive cell percentage scores showed direct correlation with tumor SBR score, whereas only positive cell percentage scores of Igκ and Igγ and not signal intensity scores were correlated with the presence of axillary lymph node metastasis. There was no significant association of Igκ and Igγ expression (considering both signal intensity and percentage) with patient age, tumor size or the expression of ER, PR, HER2/neu, p53, PCNA or nm23 (data not shown in Table 6).

**Table 6:** Multiple pairwise kendall tau-b correlations among Igκ, Igγ and other clinical indices.

	statistic	Igκ	Igκ	Igγ	Igγ	AJCC	SBR	Histotype	Axillary	Tumor
		intensity	(%)	intensity	(%)	stage	score		nodes	size
Igκ intensity	K-tau*	1	0.56	0.86	0.54	0.16	0.16	−0.29	0.05	−0.03
	P		0.00**	0.00**	0.00**	0.02**	0.02**	0.00**	0.41	0.63
Igκ**(%)**	K-tau		1	0.47	0.84	0.17	0.06	−0.43	0.14	−0.05
	P			0.00**	0.00**	0.01**	0.37	0.00**	0.02**	0.43
Igγ intensity	K-tau			1	0.57	0.12	0.18	−0.24	0.02	−0.04
	P			.	0.00**	0.07**	0.01**	0.00**	0.71	0.6
Igγ**(%)**	K-tau				1	0.15	0.05	−0.42	0.13	−0.06
	P				.	0.01**	0.43	0.00**	0.03**	0.33

* K-tau is a nonparametric measure of correlation.

** Statistically significant if *p* value <0.05.

## Discussion

By performing ISH with an IGHG1 antisense probe, we identified IGHG1 mRNA expression in breast cancer cells. Through detecting the expression of both Igγ and IGHG1 mRNA in the same breast cancer cells using ISH and IHC on serial sections we confirmed that Igγ expression in breast cancer cells was the result of production of IgG by these cells. The expression of IGHG1 mRNA in breast cancer cells nicely agrees with previous studies demonstrating IgG gene transcripts in purified breast cancer cells, breast cancer cell lines and breast cancer tissues [4], [6], [10]. Applying various cell markers we demonstrated that the IGHG1 expressing cells were indeed breast cancer cells.

Our results do not disagree with previous reports by Schmidt et al. [24] that infiltrating plasma cells can produce IgG. The difference between our study and theirs is that we observed, in addition, that the cancer cells themselves can also produce IgG. The fact that masses of cancer cells are often diffusely positive for IgG immunostaining has been disregarded frequently as background reaction or absorption of IgG from the surrounding environment. In this study, we obtained extensive evidence with a wide range of techniques to support our conclusion that cancer cells can indeed produce IgG. In particular, we used laser guided microdissection to collect cancer cells only and then detected the mRNA and the protein. We also performed *in situ* hybridization to demonstrate the abundant presence of IgG mRNA in breast cancer cells. The infiltrating B lymphocytes and plasma cells also contain IgG. Previous studies have been conducted to correlate plasma IgG levels to breast cancer development [25], [26], [27], [28]. The increased plasma IgG level could be derived from the patients' immune response or from cancer-produced IgG.

First, the growth pattern of breast cancer cells appeared to be related to IgG expression. Cancer cells arrayed in scattered small cords, small nests or individually showed more positive staining cells than those arrayed in large groups and syncytial sheets (Fig. 2, A and B).

Second, IgG expression showed correlation with malignancy of the breast diseases and histologic subtypes of carcinoma. ILC and IDC showed significantly greater Igκ and Igγ expression as compared with MC, and CIS, as well as benign lesions, including benign lesions with epithelial proliferation, such as fibroadenomas and breast hyperplasia. These results are in accordance with earlier studies on the IgG expression in benign and malignant lesions. Li et al. demonstrated a higher level of Ig κ light chain mRNA expression in cervical carcinoma than in cervical tissues with cervicitis [8]. In addition, Chen et al. found a significant difference in Igκ light chain protein expression between benign and malignant soft tissue tumors [29]. It is well recognized that the prognoses of IDC and ILC are similar [30], [31], [32], while MC is considerably less aggressive, and CIS (and of course benign lesions) are noninvasive. In view of the sharply differing clinical behavior of these neoplasms, the differing expression of IgG in IDC and ILC as compared with MC suggests the possibility that IgG expression is related to cancer cell growth. In addition, previous *in vitro* experiments showed that blockade of IgG by either antisense DNA or antihuman IgG antibody increased apoptosis and inhibited growth of epithelial cancer cells *in vitro* [6]. The results suggest that IgG expression is associated with malignancy of breast diseases and tumor-derived IgG may promote the growth and survival of cancer cells *in vivo*.

Moreover, we found that Igκ and Igγ are also positively correlated with the AJCC stage of breast cancer, SBR score and also with presence of axillary metastases. AJCC stage is assigned according to tumor size together with extent of axillary and generalized metastases. Tumor stage in this study was directly correlated with both the percentage of cells expressing Ig and the signal intensity. The SBR score in breast cancer is a microscopic index for predicting tumor behavior, based upon tumor differentiation (tubule formation), mitotic rate and nuclear grade. In this study, this score was correlated with Igκ and Igγ signal intensity, but not with the percentage of Ig positive cells. This result indicates that poorly differentiated breast cancer cells express more IgG than well differentiated breast cancer cells. This finding is in line with results by Zhang et al., who showed correlation between Igγ expression and tumor grade in esophageal squamous cell carcinomas [33]. Chen et al. also demonstrated correlation between Igκ expression and tumor grades of various soft tissue tumors [29]. AJCC stage and SBR score together are powerful tools for estimation of clinical prognosis and are typically used to guide therapy of breast cancer patients. The relationship of Ig expression to these prognostic indicators supports the concept that Ig plays a role in tumor biology of breast cancer, and may do so by influencing tumor cell growth *in vivo*. The relationship of IgG to prognosis raises the possibility that IgG expression can be used as a clinical prognostic indicator.

In this study we found no correlation of expression of Igκ and Igγ with the status of ER, PR, HER2/neu, p53, PCNA, or nm23. These markers all have association with breast tumor biology and all (except ER, PR and nm23) are positively correlated with more aggressive tumor growth. The correlation among breast cancer derived IgG, other tumor markers and tumor growth may be of different extents with varying mechanisms. Those other markers and IgG both positively associated with more malignancy does not necessarily lead to positive correlation of IgG and those other markers although the same tendency was demonstrated but no statistical significance has been reached.

Igκ and Igγ expression in this study were not related to lymphocyte infiltration in tumor tissues. All MCs showed variably lymphocytic infiltration, but these tumors showed no greater Ig expression than other tumors which lacked lymphocyte infiltration. IHC on serial sections in IDCs with infiltrating lymphocytes confirmed that Igκ positive cells were cancer cells (carcinoma cells identified by cytokeratin positivity) and not B lymphocytes (CD20 positivity in interstitial lymphocytes, not found in cancer cells)

It was of note that some normal breast tissues in this study also showed limited focal/sporadic expression of Igκ and Igγ. This result was not consistent with a previous report which suggested Ig is not expressed in normal human breast tissue [6]. However, normal human lung tissues and an embryonic renal cell line (293 cell line) have been reported to express IgG [4], [6]. This immunoglobulin expression was at a lower level than lymphocytes and plasma cells, and is of uncertain significance. In mice, IgG expression has been demonstrated in the glandular epithelial cells of lactating mammary glands [34].

Although more than 100 individual factors have been reported in the literature as independent prognostic markers in breast cancer, few of these factors have found their way into clinical application as prognostic tools. To date, ER and/or PR are the only well-defined predictive factors for response to endocrine therapy [35]. As a protein of special biologic function, Ig expressed by breast cancer or other epithelial cancers is likely to find use as prognostic marker to guide therapy, especially immunotherapy. In the last ten years, arguably the most important step in cancer therapy progress has been targeted immunotherapy. For example, the humanized monoclonal antibodies against Her 2 receptor (trastuzumab, Herceptin®) [36] and those against vascular endothelial growth factor (VEGF) a ligand for VEGF receptor 2 (bevacizumab, Avastin®) [37], have emerged as treatments for patients with advanced stages of breast cancer. Tumor antigens selected as treatment targets must be strictly evaluated to satisfy conditions such as overexpression in cancer, low or absent expression in normal tissue, and specific humoral or cellular immune response. For ovarian cancer, the first steps for the development of targeted therapy against Ig expressed by ovarian cancer cells have been taken by Lee et al. First, a specific monoclonal antibody, designated as RP215, was generated using an ovarian cancer cell line (OC-3-VGH). This monoclonal antibody is directed against an antigen, called CA215, which constitutes a carbohydrate associated epitope of Ig heavy chains. This epitope is present in Igs expressed by (ovarian) cancer cells but not in normal Igs produced by specialized B lymphocytes [38]. Second, experiments with nude mice bearing ovarian tumors showed that intraperitoneal injection with RP215 antibodies resulted in significantly decreased tumor size compared with untreated mice [39]. Third, chimerization of RP215 antibodies was performed [39]. It is noteworthy that additional cancer cell lines and tissues of other cancer types, including breast cancers, were also found to express CA215 [39], [40]. The extensive work of Lee et al. showed that CA215 can be used as a therapeutic target. RP215 is a monoclonal antibody against CA215 which is a mixture of immunoglobulin superfamily heavy chain-like molecules (mostly IgG) of which the effective epitope contains a carbohydrate moiety and has been used as a pan cancer marker but not detectable in B lymphocytes/plasma cells [41]. However, the IgG we detected was also present in B lymphocytes/plasma cells.

The carcinogenic process of breast cancer emerges from the evolution of atypical ductal hyperplasia into ductal carcinoma in situ (DCIS), and then progresses into invasive breast cancer [42] and metastasis. The mechanisms driving those processes are not clear, but evidence suggests that a succession of molecular event leads to different phenotypes or heterogeneity of breast cancer [43]. The expression patterns of IgG observed in the study showed an increasing tendency from normal, benign, moderate to highly malignant cancer types as well as correlating to different subtypes and AJCC stages, with more malignant cancer cells/types having more abundant IgG expression. Additionally, within the same case of IDC, more abundance of IgG expression were observed in infiltrating cancer cells than in large cancer nests. The study of this cohort of 186 breast cancers, 20 benign breast lesions and 30 normal breast tissues plus our recent comparative investigation of another 68 cases of metastatic and non-metastatic breast cancers [14] suggest that IgG producing cancer cells might have more aggressive behavior. This phenomenon may provide a valuable indicator for evaluation of clinical behavior and prognosis of breast cancer. Functional significance of this newly discovered phenomenon in breast cancer is indicated.
